# Gentiopicroside targeting AKT1 activates HIF-1α/VEGF axis promoting diabetic ulcer wound healing

**DOI:** 10.3389/fphar.2025.1506499

**Published:** 2025-02-26

**Authors:** Xinxia Wang, Mingyan Liu, Yao Wu, Jianguo Sun, Li Liu, Zheng Pan

**Affiliations:** ^1^ Department of Pharmacy, Shanghai Jiahui International Hospital Pharmacy, Shanghai, China; ^2^ Department of Opreating Room, Obstetrics and Gynecology Hospital of Fudan University, Shanghai, China; ^3^ Department of Otolaryngology, 980th Hospital of The Joint Logistics Support Force, Shijiazhuang, Hebei, China; ^4^ Department of Pharmacy, Second Affiliated Hospital of Naval Medical University, Shanghai, China; ^5^ College of Traditional Chinese Medicine, Chongqing Medical University, Chongqing, China

**Keywords:** gentiopicroside, diabetic ulcer, wound healing, CETSA assay, geo data, HIF-1α/VEGF axis

## Abstract

**Backgound:**

Gentiopicroside (GSP) have been proven to accelerate the healing of diabetic ulcers (DU), but the underlying molecular mechanisms remain unclear. This study aims to explore the mechanism by which GSP accelerates the healing of DU.

**Method:**

The targets of GSP were firstly predicted using the SuperPred, SwissTargetPrediction, and Pharmmapper databases; DU-related transcriptome data were obtained from the GEO database, including GSE147890, GSE68183, and GSE199939; differential expression analysis was conducted using the Limma package, and DU-related targets were identified after summarization and de-duplication. Then, Potential targets for GSP treatment of DU were screened by Venn analysis; core targets for GSP treatment of DU were selected by constructing a protein-protein interaction (PPI) network; the mechanism of GSP treatment of DU was predicted by GO and KEGG enrichment analysis. Finally, the target binding of GSP to core targets was evaluated by molecular docking and CETSA assay, and *in vitro* experiments were conducted using L929 cells to validate the findings.

**Result:**

A total of 538 targets of GSP and 10795 DU-related targets were predicted; Venn analysis identified 215 potential targets for GSP to accelerate DU wound healing; PPI network analysis suggested that AKT1 may be core targets for GSP treatment of DU; GO and KEGG enrichment analysis showed that pathways such as HIF-1 and VEGF are closely related to the treatment of DU with GSP, and it also participates in the regulation of various biological processes such as small molecule catabolism and leukocyte migration to exert its therapeutic effect on DU. Molecular docking and CETSA detection indicated that GSP can target bind to AKT1. The experimental results confirmed that GSP can significantly promote the proliferation and migration of L929 cells. Westen Blot results showed that GSP can accelerate DU wound healing via AKT1/HIF-1α/VEGF axis.

**Conclusion:**

GSP target binding to AKT1 accelerates DU wound healing via the regulation of HIF-1α/VEGF axis.

## 1 Introduction

Diabetes mellitus is a serious chronic metabolic disease mainly featured by hyperglycemia, with a high prevalence. According to authoritative forecasts, the global prevalence of diabetes will reach 9.9% by 2045 (Umpierrez et al., 2024). Persistent hyperglycemia can cause many complications (Sathyaraj et al., 2023), of which the most common complication is diabetic ulcer (DU). DU is mainly manifested as slow wound healing or even non-healing symptoms, and the treatment is very challenging; DU is easy to further lead to disability or even death if timely intervention is not carried out, which causes a heavy economic burden and great psychological pressure on the patients (Ng et al., 2021; Sharma et al., 2023; Veith et al., 2019). In addition, when diabetic patients are complicated with malignant tumors and other diseases that require surgical intervention, the wound caused by surgery may also slow to heal and even lead to DU (Akiboye and Rayman, 2017; Grant and Chowdhury, 2022; Sykara et al., 2022), which seriously impedes the postoperative recovery, and at the same time places higher demands on postoperative treatment and care.

It is well known that wound healing is a complex dynamic process that overlaps in space and time (Theocharidis et al., 2020), including four processes: hemostasis, inflammation, proliferation and remodeling (Wilkinson and Hardman, 2020); Diabetes impedes wound healing by complex mechanisms. It has been reported that a high-glycemic environment in wounds of diabetic patients promotes apoptosis of fibroblasts and epidermal cells, thus slowing wound healing. Some also reported that diabetic environments can modulate macrophage M1 polarization and exacerbate inflammation, which impairs the wound healing process by prolonging the inflammatory phase of wound healing (Bolton, 2022; Swoboda and Held, 2022). In summary, promoting angiogenesis and modulating inflammation are the main strategies for treating DU (Sawaya et al., 2020; Yusuf Aliyu and Adeleke, 2023). Broad-spectrum antibiotics with infrared irradiation therapy is currently a common option for patients with DU and postoperative treatment of diabetic surgical patients (Wan et al., 2021). However, the toxicity and side effects of antibiotics and the susceptibility to drug resistance are debatable (Patel et al., 2019), In addition, infrared irradiation therapy has a wide inter-individual variability, the amount of red light irradiation is difficult to control, and a comprehensive standard system has not yet been established (Busanello-Costa et al., 2023). Studies have also reported the use of recombinant human epidermal growth factor, hyperbaric oxygen therapy, nitric oxide therapy, and vascular endothelial stem cells for the treatment of DU (Mavrogenis et al., 2018; Rai et al., 2022; Zheng et al., 2023; Shi et al., 2022); Besides, a large number of bioscaffolds, nanomaterials, and hydrogels have been used for the treatment of DU (Zarei et al., 2018). Despite that many approaches and strategies have been developed over the past decades, only a few therapeutic approaches have been used in the clinical treatment of DU, and their efficacy is limited (Huang J. J. et al., 2020; Rodrigues et al., 2019). There are no better therapeutic agents available for the treatment of DU; thus, there is an urgent need to develop safer and more effective therapeutic agents for DU.

Gentiopicroside (GSP) is an iridoid glycoside isolated from Gentiana macrophylla and the main active metabolite of Gentiana macrophylla (Wu et al., 2017); Gentiana macrophylla has been documented in traditional Chinese medicine for thousands of years, and there is no documented toxicity of the drug (Liu et al., 2023). Current research on GSP focuses on its anti-inflammatory activity (Zhang et al., 2018). For example, Zhang et al. demonstrated strong anti-inflammatory activity of GSP at a concentration of 100 ug/mL in the LPS-stimulated RAW264.7 cell model (Zhang et al., 2019), a result that was confirmed by Wang et al. and other studies (Wang et al., 2020). Many studies have also reported the therapeutic potential of GSP in promoting wound healing (Antoniadi et al., 2023), for example, Öztürk et al. showed that GSP promotes the mitogenic capacity of chicken embryonic fibroblasts, promotes cell proliferation, and shows good therapeutic potential for wound healing (Oztürk et al., 2006). Notably, the bioavailability of GSP administered orally is low; therefore, May Almukainzi et al. designed a wound dressing containing GSP for transdermal administration, which showed that GSP could accelerate DU wound healing in diabetic rats by inhibiting wound inflammation (Almukainzi et al., 2022a; Almukainzi et al., 2022b). Recently, it has been reported that GSP can show favorable therapeutic effects in diabetic mice and DU by improving glycolipid metabolism in diabetes (Xu et al., 2024). Although existing studies have revealed that GSP may be a potential therapeutic drug for DU, the mechanism of action of GSP in treating DU is unclear. It is necessary to further elucidate the molecular mechanism by which GSP accelerates wound healing in DU.

In this study, we analyzed the potential targets and mechanisms of GSP in treating DU through network analysis and molecular docking, and verified them by constructing a DU cell model *in vitro* for CCK-8, CETSA, and WB experiments. It showed that GSP can target and bind to AKT1 to accelerate DU wound healing through regulating the HIF-1α/VEGF axis, which provides more evidence support for GSP in treating DU.

## 2 Materials and methods

### 2.1 Reagents

GSP reference standard (batch no. MUST-23031310, purity >97%) was purchased from Chengdu Must Bio-technology Co., Ltd.; RPMI1640 cell culture medium (batch no. PWL021-240415) was purchased from Dalian Meilun Biotechnology Co., Ltd.; fetal calf serum (batch no. GA230622) was purchased from Gibco Life Sciences in the United States; CCK-8 reagent kit (batch no. Byt-10314), SDS-PAGE precast gel (batch no. Byt-0053A), RIPA Lysis Buffer (batch no. Byt-0013C), GAPDH antibody (batch no. AF1186), HPR-labelled secondary antibody (batch no. A0208) were purchased from Shanghai Beyotime Biotechnology Co., Ltd.; VEGF antibody (batch no. sc-57496), HIF-1α antibody (batch no. sc-13515), AKT1 antibody (batch no. sc-5298) and p-AKT1 antibody (5.Ser 473) (batch no. sc-293125) were purchased from Santa Cruz Biotechnology; All primary antibodies were diluted at a ratio of 1:1500 using antibody dilution buffer, and secondary antibodies were diluted at a ratio of 1:4000.

### 2.2 Screening of potential targets for GSP to accelerate DU wound healing

The chemical structure data of GSP was downloaded from Pubchem database, and then SuperPred, SwissTargetPrediction and Pharmmapper databases were used to predict the potential targets of GSP. After standardizing and compiling the targets using the UniProt database, duplicates were removed to obtain the targets of GSP.

Transcriptomics data of DU (GEO ID: GSE147890) were obtained from the GEO database, which includes transcriptomic data from skin tissues of seven control mice and six diabetic mice, as reported in the previous study. The data were divided into control and diabetic groups. Subsequently, the expression profiles were annotated using GPL571 probe data, and screened for differentially expressed genes (DEGs) with *p* < 0.05 and FoldChange >1.5 using the Limma package. In addition, two datasets of DU (GEO ID: GSE68183 and GSE199939) were collected by the same method for supplement; the DEGs of the three datasets were pooled to remove the duplicates and then the DU-related targets were obtained. Finally, the intersection was taken by Venn analysis to obtain the potential targets of GSP in accelerating DU wound healing.

### 2.3 Construction of protein-protein interaction (PPI) network

The potential targets were imported into the STRING database, specifying the species as human and setting the confidence level to 0.7. Subsequently, the PPI network information was extracted by removing isolated protein nodes and this network information was then imported into Cytoscape software for visualization. The PPI network was also analyzed by clustering using the CytoHubba plug-in, and the top 10 targets were selected as the core targets of GSP through sequencing by the MCC algorithm for the treatment of DU. Finally, the results were visualized using the ggplot2 package.

### 2.4 GO and KEGG enrichment analysis of potential targets

Integration of GO and KEGG enrichment analysis is a common method to analyze the functional information and pathway mechanisms of targets. To analyze the biological processes and signaling pathways associated with the potential targets of GSP and elucidate the mechanism of GSP in the treatment of DU, GO and KEGG enrichment analyses of potential targets were performed using the Cluster Profiler package with *p* < 0.05, with the enrichment of at least 3 targets in each pathway as the screening conditions. Finally, TOP20 KEGG pathways and TOP30 GO terms were presented using the ggplot2 package.

### 2.5 Molecular docking

In order to evaluate the interaction and binding between GSP and the core target AKT1, the protein structure file of the core target (PDB ID: 4GV1) was obtained from the PDB database and pre-docking pre-processing such as dehydrogenation and hydrogenation was performed on the core target by using the PyMol 2.5.2 software. CASTp 3.0 program was used to analyze and determine the binding pockets of the core target structure that are suitable for molecular docking, followed by molecular docking using AutoDock Vina software and finally visualization using PyMol 2.5.2 software.

### 2.6 Cellular thermal shift assay (CETSA)

CETSA is a common method used to assess receptor-ligand binding and is widely used to assess drug and protein binding. L929 cell suspension was prepared using RIPA Lysis Buffer containing protease inhibitors and then divided into control and experimental groups, which were incubated with 100 μM GSP for 2 h. After centrifugation, the cells were resuspended with PBS, after which the suspension was heated at 6 different temperature points set between 35°C–60°C. Then, the supernatant was obtained after centrifugation at 17400 g for 20 min, followed by 12% SDS-PAGE. After the end of the process, the membrane was transferred to a PVDF membrane and closed with 5% defatted goat’s milk, after which, the membrane was added to the AKT1 primary antibody for incubation at 4°C overnight. Subsequently, the membrane was washed sufficiently with TBST and then added secondary antibody to incubate at room temperature for 2 h. TBST was added to ECL luminescent solution for development after the second washing of the membrane, and the results were quantified using ImageJ software and statistically analyzed and visualized using GraphPad Prism 9.0.

### 2.7 Cell culture

L929 cells were purchased from ORiCells Biotechnology (Shanghai) Co., Ltd. and cultured in complete medium supplemented with 10% fetal calf serum, 1% penicillin-streptomycin double antibody, and 89% RPMI-1640 medium; all cellular experiments were performed without *mycoplasma* contamination. Referring to previous studies (Huang X. et al., 2020; Li et al., 2021), high glucose medium for culturing L929 cells was prepared by adding an additional 50 mM glucose and 100 μM palmitic acid to complete medium for the preparation of DU cell models. The incubator environment was 37 °C with a humid constant temperature containing 5% CO_2_, and cell passaging was performed when cells grew to 80% confluence.

### 2.8 CCK-8 assay

The effect of GSP on the proliferation of L929 cells was assessed using CCK8 assay. Firstly, L929 cells in the logarithmic growth phase were seeded into 96-well plates at a density of 3*103 cells/well, and the cells were divided into control, model and experimental groups. Except for the control group, the cells of each group were cultured with high glucose medium, and the experimental group was treated with different concentrations of GSP. After 24 h, each well was incubated in the incubator by adding 10 μL of CCK-8 working solution for 2 h, and then the absorbance of each well at 450 nM was detected under an enzyme marker and the results were calculated.

### 2.9 Wound healing assay

L929 cells in the logarithmic growth phase were taken and seeded in 6-well plates at 5*10^4^ cells/well, and the grouping and drug administration were the same as in **2.8**, and concentrations of GSP were set as100 μM and 200 μM (Group of GSP-L and GSP-H). After the cells had grown all over the Petri dish, a disposable lance tip was used to scratch the cells. Subsequently, the drug-containing medium with different concentrations of GSP was added after the cell debris had been rinsed repeatedly using PBS and the cells were observed for cellular healing at 0, 8, 24, and 48 h. The results were statistically analyzed using ImageJ software.

### 2.10 Western blotting

L929 cells in the logarithmic growth phase were taken and seeded in 6-well plates at 5*10^4^ cells/well, the cell grouping and administration were the same as in **2.9**. Then, total protein was extracted using RIPA Lysis Buffer and protein concentration was quantified by BCA method. Protein samples were subjected to SDS-PAGE, transferred to PVDF membrane, closed by adding 5% defatted milk for 1 h, then added primary antibody and incubated at 4 °C overnight, and washed three times with TBST; they were added secondary antibody incubated at room temperature for 2 h, washed three times with TBST, and added ECL luminescent solution for development imaging. The results were normalized by GAPDH as internal reference.

### 2.11 Statistical analyses

All data were processed using GraphPad Prism and ImageJ software. Differences were calculated by t-test for comparisons between two groups, and comparisons between multiple groups were assessed by one-way ANOVA. The data were presented in the form of Mean ± SEM. *P* < 0.05 was considered statistically significant, * indicates *P* < 0.05; ** indicates *P* < 0.01; *** indicates *P* < 0.001; **** indicates *P* < 0.0001.

## 3 Results

### 3.1 Potential targets for GSP to accelerate DU wound healing

**FIGURE 1:**
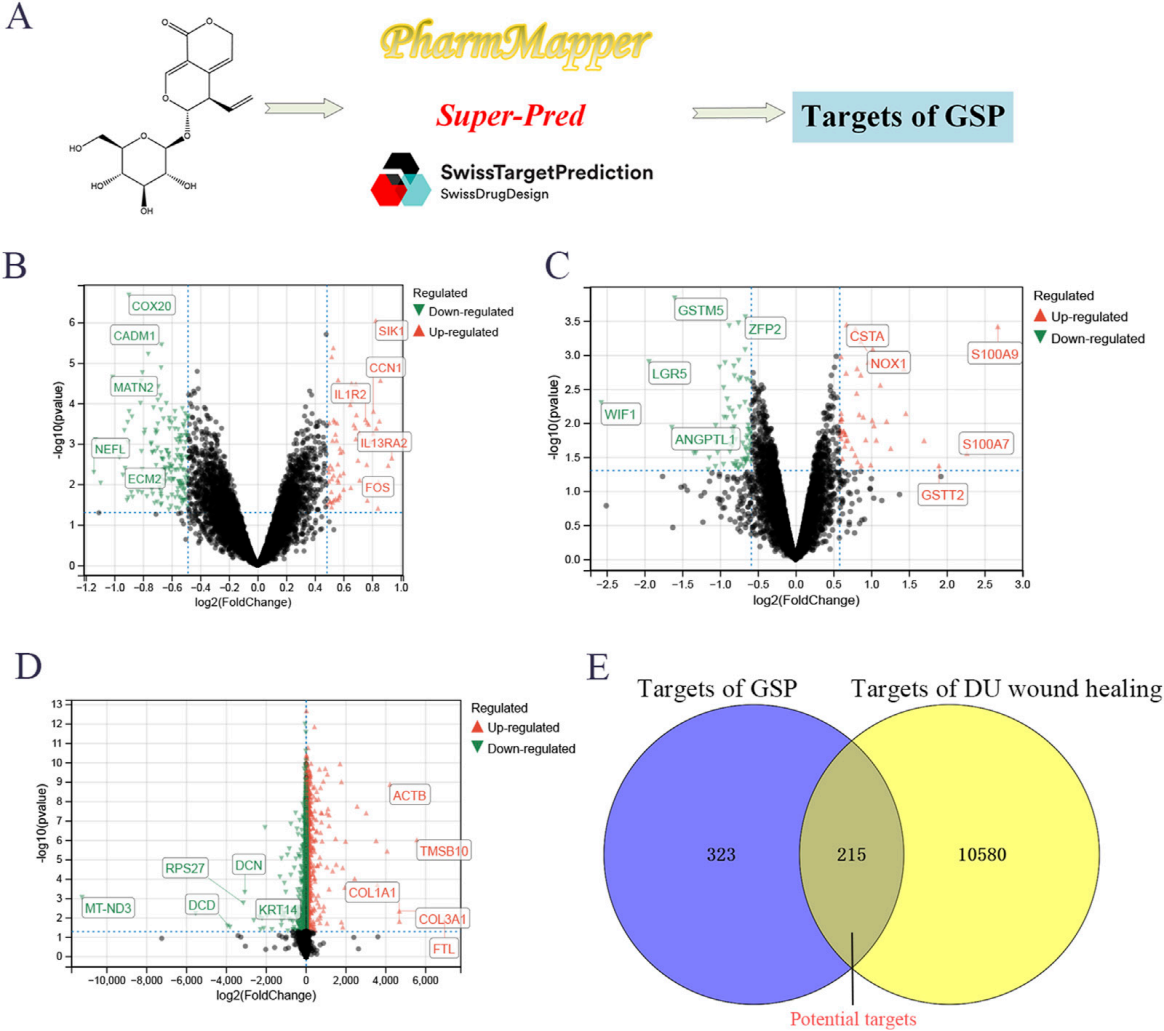
Screening targets of Gentiopicroside **(A)**. The differential expression analysis to screen the targets of DU, including GSE147890 **(B)**, GSE68183 **(C)**, and GSE199939 **(D)**. Venn analysis between gentipicroside targets and DU targets **(E)**.

A total of 538 targets of GSP were collected through SuperPred, SwissTargetPrediction and Pharmmapper databases; the variance analysis of three datasets, GSE147890, GSE68183 and GSE199939, identified a total of 10,795 DU-associated targets (Figures 1A–C). Venn analysis showed a total of 215 intersecting targets between drug and disease targets (Figures 1D, E), suggesting that GSP may accelerate DU wound healing through these 215 potential targets.

### 3.2 Core targets for GSP to accelerate DU wound healing

**FIGURE 2:**
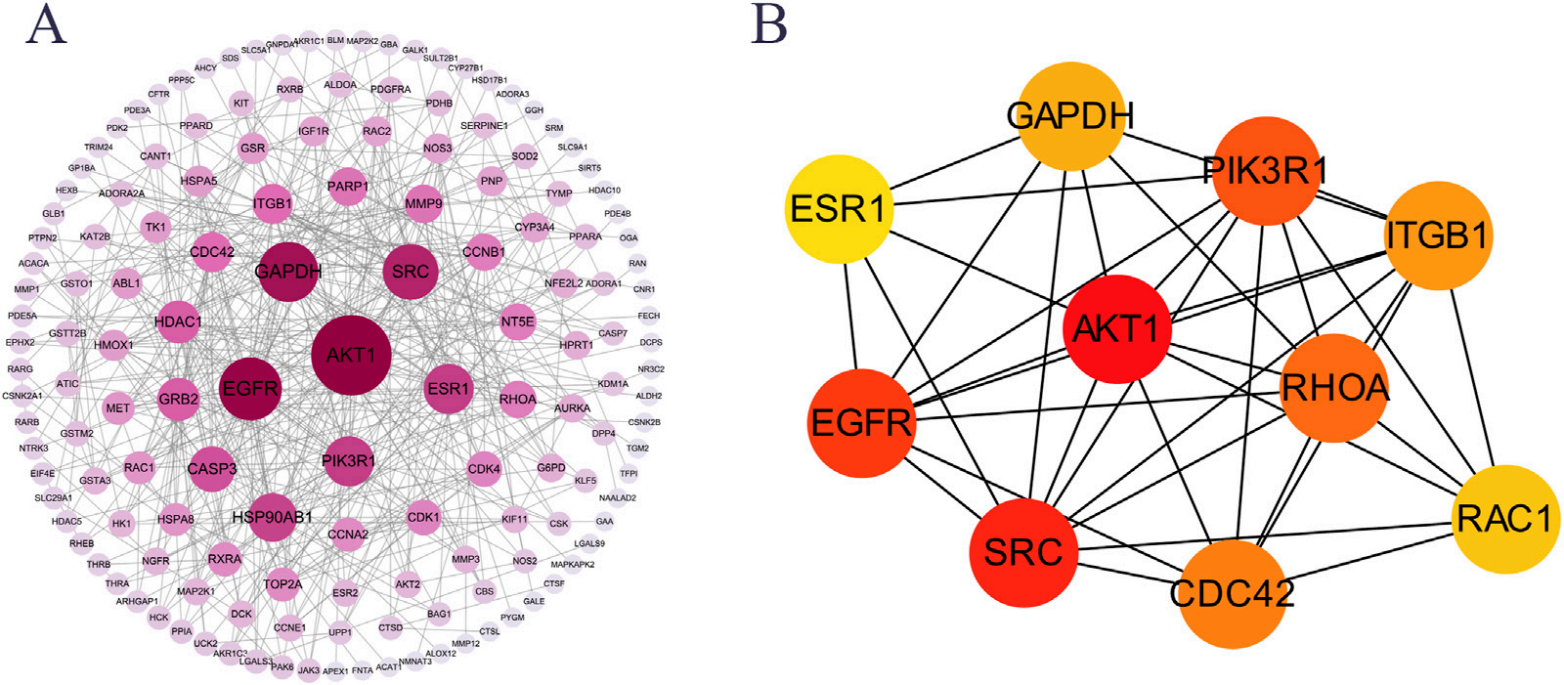
PPI network analysis of the potential targets. **(A)** PPI network. **(B)** Core targets were screen out by Cytohubba plugin.

PPI network analysis was performed on 215 potential targets using the STRING database and Cytoscape 3.9.1 software, and cluster analysis was performed on the PPI network using the Cytuhubba plug-in; the results showed that the core targets of GSP for DU treatment were mainly AKT1, SRC, EGFR, PIK3R1, RHOA, CDC42 and ITGB1 (Figure 2A). Calculated by Cytohubba plug-in and ranked by MCC algorithm, the results showed that AKT1 scored 4130 and ranked first, therefore, AKT1 may be the core target of GSP for DU treatment (Figure 2B).

### 3.3 GO and KEGG enrichment analysis

**FIGURE 3:**
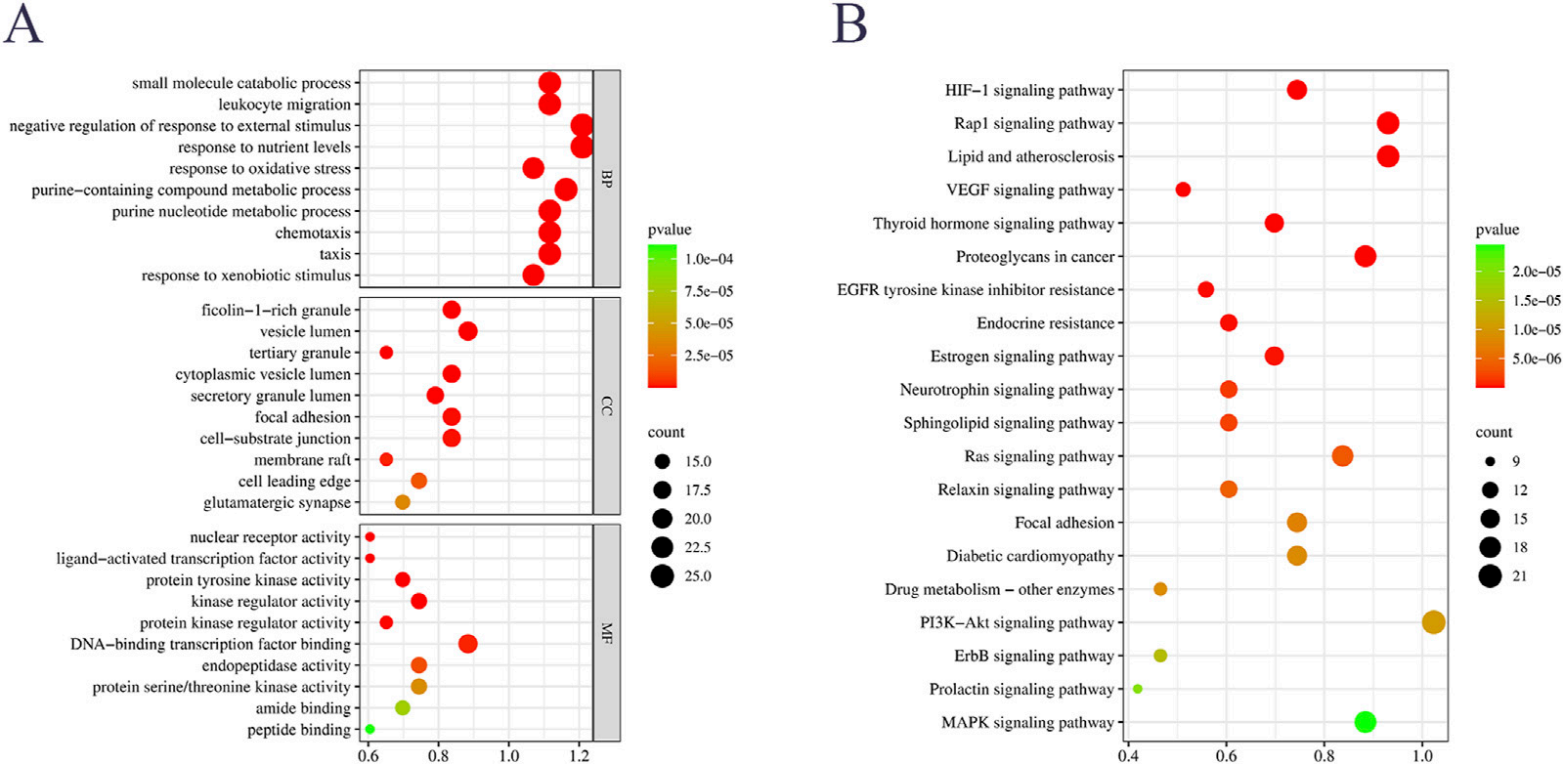
GO **(A)** and KEGG **(B)** enrichment analyses of potential targets.

The items obtained from the GO enrichment analysis of potential targets included 1203 biological processes (BP), 49 cellular components (CC) and 121 molecular functions (MF). Among them, the major biological processes included small molecule catabolic processes, leukocyte migration, negative regulation of responses to external stimuli and responses to nutrient levels, etc. The CC included ficolin-1-rich granules, vesicular lumen, tertiary granules and cytoplasmic vesicle lumen, etc., and the molecular functions included nuclear receptor activity, ligand-activated transcription factor activity, protein kinase-regulated activity, and kinase-regulated activity, etc. (Figure 3A). These results suggested that GSP may exert therapeutic effects on DU by modulating biological processes such as small molecule catabolic processes and leukocyte migration.

The KEGG enrichment analysis of the potential targets identified 120 signaling pathways, and the results showed that the potential targets of GSP in treating DU mainly involved the signaling pathways of HIF-1, Rap1 and VEGF, etc. (Figure 3B). Referring to previous studies, it has been reported that the HIF-1 pathway is upstream of the VEGF pathway, and plays a role in regulating the VEGF pathway (Rattner et al., 2019; Zhang et al., 2023). Therefore, combined with the results of KEGG enrichment analysis, we speculate that GSP may play a therapeutic role in DU through the HIF-1α/VEGF axis.

### 3.4 GSP could target bind to AKT1

**FIGURE 4:**
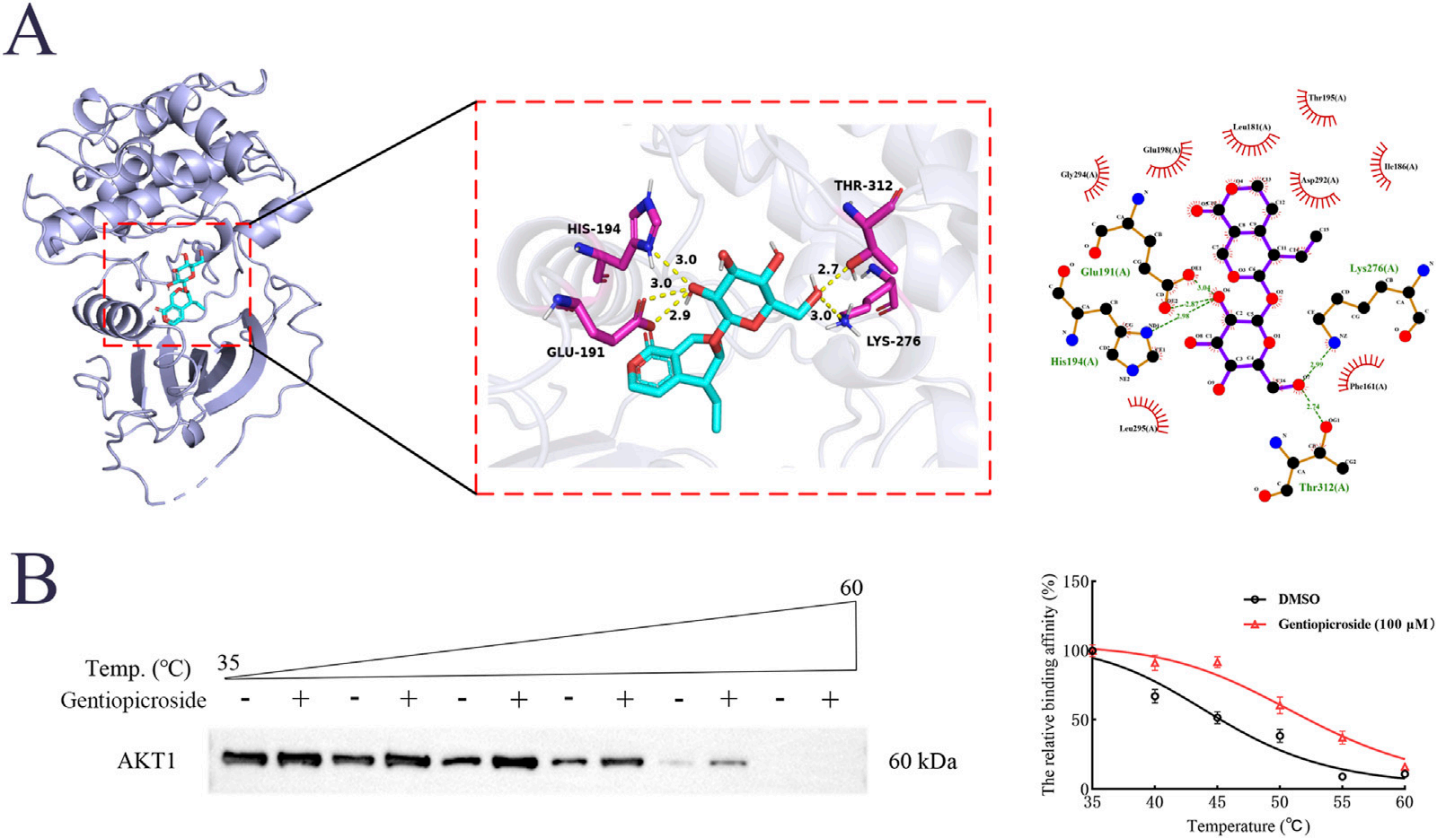
Molecular docking analysis **(A)** and CETSA assay **(B)** of gentiopicroside binding to AKT1.

The molecular results showed that the binding energy of GSP to AKT1 was −8.1 kcal/mol, which indicated that GSP could bind to AKT1 to form a stable complex. The docking of GSP and AKT1 was then visualized using PyMOL, which showed that GSP and AKT1 could form hydrogen bonds at HIS-194, GLU-191, THR-312 and LYS-276 (Figure 4A). In addition, lower thermal degradation of AKT1 protein after GSP intervention was observed by CETSA experiments, and the CETSA curve was shifted to the right, which indicated that AKT1 protein was more thermally stable after GSP intervention (Figure 4B). It suggests that there could be targeted binding between GSP and AKT1. These results indicate that GSP may accelerate DU wound healing by binding to AKT1.

### 3.5 GSP could promote high glucose-induced L929 cell proliferation and migration

**FIGURE 5:**
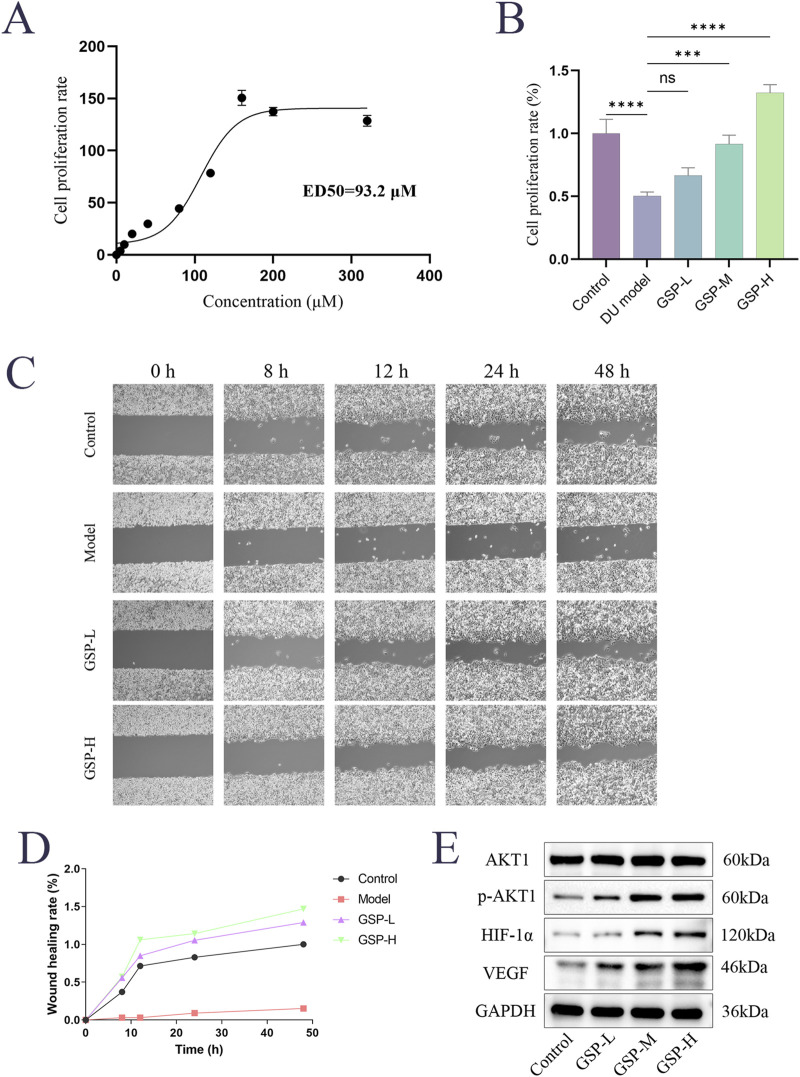
Gentiopicroside can promote the proliferation and migration of L929 cells in high serum glucose and serum lipid environment. **(A, B)** CCK-8 assay. **(C, D)** Wound healing assay. **(E)** The effect of GSP on key proteins.

The effects of GSP on high glucose-induced L929 cell proliferation were examined by CCK8 assay. The results showed that cell proliferation was significantly decreased in the DU model group, and the GSP intervention promoted cell proliferation and showed a dose-dependence within a certain range; the proliferation rate of L929 cells reached the maximum when the concentration of GSP reached 160 μM, and the ED_50_ of GSP is 93.2Μm (Figures 5A, B). In addition, the effect of GSP on high glucose-induced migration of L929 cells was also explored by wound healing assay. The results showed that GSP significantly promoted L929 cell migration, and the wound healing rate was significantly higher than the DU model group (Figures 5C, D); these results are consistent with previous studies, suggesting that GSP may be a potential therapeutic drug for DU, and can significantly accelerate the rate of DU wound healing.

### 3.6 GSP exerts therapeutic effects on DU via the HIF-1α/VEGF axis

We noted from the results of KEGG enrichment analysis that HIF-1 and VEGF signaling pathways may play an important role in the acceleration of DU wound healing by GSP. A large number of previous papers reported an important role of the HIF-1α/VEGF axis in the modulation of angiogenesis. Based on that, we speculated that targeted binding to AKT1 by GSP may accelerate DU wound healing through the regulation of the AKT1/HIF-1α/VEGF axis. The expression levels of AKT1, p-AKT1, VEGF and HIF-1α in L929 cells were measured by Westen Blot; the results showed that GSP significantly increase the phosphorylation level of AKT1, whereas there was no significant change in the total protein AKT1, and the expression of VEGF and HIF-1α also increased (Figure 5E). These results suggest that targeted binding to AKT1 by GSP can accelerate DU wound healing by activating the AKT1/HIF-1α/VEGF axis, further confirming the predicted results of network analysis.

## 4 Discussion

DU is the most common complication of diabetes mellitus. The main manifestation of DU patients is slow wound healing or even non-healing, and untimely intervention may even lead to amputation, which seriously affects the quality of life and prognosis of diabetic patients (Simões et al., 2022). Moreover, when diabetic patients with other complications require surgical treatment, the trauma caused by surgery is very likely to induce DU, and further cause ulcerative pressure ulcers and secondary infections, which seriously threaten the life of diabetic patients (Liu et al., 2022); This puts high demands on the postoperative treatment and care of patients with diabetic surgery and is an important challenge that needs to be urgently solved at present. Currently, the first line of wound healing treatment for diabetic patients is broad-spectrum antibiotics combined with infrared irradiation, but this method has many limitations, such as the toxic side effects of antibiotics and the easy formation of drug resistance, which are still debatable (Patel et al., 2019; Wan et al., 2021), Studies have shown that infrared irradiation therapy has a large inter-individual variability, and it is difficult to control the red light irradiation, so a standard system has not yet been formed (Busanello-Costa et al., 2023). This poses a great challenge for the treatment of DU, so it is necessary to develop related drugs and therapies.

Previous studies have demonstrated that GSP’s hydrogel wound dressing has favorable therapeutic effects on DU wound healing (Almukainzi et al., 2022a; Almukainzi et al., 2022b), and some studies have also reported that GSP can exhibit favorable therapeutic effects in diabetic mice and DU by improving glycolipid metabolism in diabetes (Xu et al., 2024). These studies suggest the therapeutic potential of GSP in DU. The study aimed to validate the target and key mechanism of GSP in accelerating DU wound healing through network analysis, molecular docking, CETSA and *in vitro* experiments, and the results showed that GSP could target and bind to AKT1 to activate the AKT1//HIF-1α/VEGF axis and accelerate the wound healing in DU, providing more evidence for further research and application of GSP in the treatment of DU. Interestingly, in this study, GSP was found to upregulate the phosphorylation level of AKT1 in L929 cells, a previous study has also demonstrated that GSP can target PAQR3 to activate the PI3K/AKT pathway and restore the insulin signaling pathway (Xiao et al., 2022), suggesting that GSP may be a potential candidate for diabetes. In conclusion, a large number of literature have confirmed that upregulation of the AKT1/HIF-1α/VEGF axis accelerates wound healing by promoting angiogenesis, consistent with our findings (Wang et al., 2024; Wang et al., 2014). Our results suggest that GSP promotes the phosphorylation of AKT1 after targeting and binding to it, thus activating the downstream HIF-1 pathway and upregulating VEGF to accelerate angiogenesis.

This study elucidates the mechanism of GSP accelerating DU wound healing, but there are still many limitations to further study. First, considering that GSP has been previously demonstrated to be effective in the treatment of DU *in vivo* in previous studies (Almukainzi et al., 2022b; Li et al., 2024), *in vivo* validation was not performed in this study. However, it is worth noting that GSP, as a secoiridoid, is easily metabolized *in vivo*, resulting in its low bioavailability, so the route of administration of GSP is very important. At present, transdermal administration of hydrogel dressing is a common way of GSP for the treatment of DU, but the problems of allergic reaction, pain of dressing change, convenience, and infection due to untimely dressing change indicate that there are many drawbacks of transdermal administration of hydrogel (Matoori et al., 2021; Yang et al., 2021; Zhao et al., 2024), so the route of administration of GSP in the treatment of DU should be further improved in subsequent studies. In addition, this study only determined that GSP can target bind AKT1 by CETSA assay, but this result needs to be supported by more evidence. Finally, follow-up studies are necessary to further explore more targets and mechanisms of GSP in accelerating DU wound healing, so as to provide more data support for the application of GSP.

## 5 Conclusion

This study demonstrated that AKT1 might be the core target of GSP to accelerate DU wound healing by network analysis and molecular docking, and CETSA assay elucidated that GSP could bind to AKT1 tightly. The results of KEGG enrichment analysis showed that the HIF-1 and VEGF pathways were the key pathways of GSP for DU treatment, and *in vitro* validation was performed. In conclusion, our results indicate that GSP can target bind to AKT1 to activate the HIF-1α/VEGF pathway and accelerate DU wound healing, which provides supportive data for the further application of GSP in DU treatment.

## Data Availability

The original contributions presented in the study are included in the article/supplementary material, further inquiries can be directed to the corresponding authors.
